# Everything but the Kitchen Sink: An Analysis of Bacterial and Chemical Contaminants Found in Syringe Residue From People Who Inject Drugs

**DOI:** 10.1093/ofid/ofad628

**Published:** 2023-12-11

**Authors:** John A Wildenthal, Drew J Schwartz, Nathanial S Nolan, Lingxia Zhao, John I Robinson, Erin Jones, Raagini Jawa, Jeffrey P Henderson, Laura R Marks

**Affiliations:** Medical Scientist Training Program, Washington University in St Louis School of Medicine, St Louis, Missouri, USA; Department of Pediatrics, Division of Infectious Diseases, Washington University in St Louis School of Medicine, St Louis, Missouri, USA; Department of Molecular Microbiology, Washington University in St Louis School of Medicine, St Louis, Missouri, USA; Division of Infectious Diseases, Department of Internal Medicine, Washington University in St Louis School of Medicine, St Louis, Missouri, USA; Division of Infectious Disease, Department of Internal Medicine, Veterans Affairs St Louis Health Care, St Louis, Missouri, USA; Department of Molecular Microbiology, Washington University in St Louis School of Medicine, St Louis, Missouri, USA; Department of Molecular Microbiology, Washington University in St Louis School of Medicine, St Louis, Missouri, USA; Division of Infectious Diseases, Department of Internal Medicine, Washington University in St Louis School of Medicine, St Louis, Missouri, USA; Department of Molecular Microbiology, Washington University in St Louis School of Medicine, St Louis, Missouri, USA; Center for Research on Healthcare, Division of General Internal Medicine, University of Pittsburgh School of Medicine, Pittsburgh, Pennsylvania, USA; Department of Molecular Microbiology, Washington University in St Louis School of Medicine, St Louis, Missouri, USA; Division of Infectious Diseases, Department of Internal Medicine, Washington University in St Louis School of Medicine, St Louis, Missouri, USA; Division of Infectious Diseases, Department of Internal Medicine, Washington University in St Louis School of Medicine, St Louis, Missouri, USA

**Keywords:** *Bacillus cereus*, fentanyl, injection drug use, *Staphylococcus aureus*, xylazine

## Abstract

**Background:**

People who inject drugs (PWID) are at high risk of severe wounds, invasive infections, and overdoses. To date, there are few data on the bacterial and chemical contaminants PWID are exposed to when using illicitly manufactured fentanyls and stimulants.

**Methods:**

Previously used injection drug use equipment was recovered in St Louis, Missouri, by harm reduction organizations over a 12-month period. Syringe residue was analyzed for bacterial contaminants by routine culturing followed by whole genome sequencing of single bacterial isolates. Chemical adulterants in syringe residue were identified by liquid chromatography–mass spectrometry.

**Results:**

Bacteria were cultured from 58.75% of 160 syringes analyzed. Polymicrobial growth was common and was observed in 23.75% of samples. *Bacillus cereus* was the most common pathogen present and was observed in 20.6% of syringe residues, followed closely by *Staphylococcus aureus* at 18.8%. One hundred syringes underwent mass spectrometry, which demonstrated that chemical adulterants were common and included caffeine, diphenhydramine, lidocaine, quinine, and xylazine.

**Conclusions:**

Analysis of syringe residue from discarded drug use equipment demonstrates both chemical and biological contaminants, including medically important pathogens and adulterants.

## Teaser

Analysis of syringe residue of discarded drug use equipment demonstrates a high prevalence of both chemical and biological contaminants, including medically important pathogens.

Most overdose deaths in the United States (US) involve illicitly manufactured fentanyls, heroin, cocaine, or methamphetamine (either alone or in combination) [1, 2]. Due to their unregulated nature, the majority of illicit drugs are manufactured, stored, and distributed in nonsterile conditions, potentially facilitating the introduction of bacterial pathogens and contamination with chemical adulterants. The result is a surging syndemic of overdoses, severe wounds, and invasive infections among people who use drugs. People who inject drugs (PWID) in particular are at grave risk of a range of infectious complications, with up to 68% [3] experiencing the spectrum of minor skin and soft tissue infections to life-threatening endovascular infections [4].

Data on the microbial diversity of bacterial pathogens causing infections among PWID is generally limited to epidemiologic trends of infective endocarditis in populations with opioid use disorder or hepatitis C virus [5, 6], or small case series [7]. Outbreaks of harmful pathogens linked to illicit drugs have ranged from uncommon pathogens such as *Bacillus anthracis* [8] to more ubiquitous organisms such as *Staphylococcus aureus* [9–11]. Relatively little information exists on the potential sources of most infections among PWID, and data on the risk posed by bacterial contamination of drugs or drug use equipment is lacking. The existing literature is limited to several small studies on the microflora of heroin conducted in the 1970s and 1980s [12–14] and again in 2002 [15]. These previous reports documented high levels of bacterial contaminants; however, over the subsequent 2 decades the risk of bacterial infection from the drug supply itself has been largely ignored. Interestingly, heroin as well as quinine (a common heroin adulterant) was reported to have limited antibacterial properties [16]. Public health messaging on infection prevention has therefore focused almost exclusively on the risk posed by reuse and licking of needles and failure to clean skin sites [17]. With the ever-increasing number of infectious complications occurring among PWID, it is important to understand how prior data on bacterial contamination of heroin samples relate 2 decades later to the current US illicit drug supply that is composed largely of illicitly manufactured fentanyls and stimulants.

Similarly, there is also increasing awareness of the potential for novel chemical adulterants within the illicit drug supply that may harm consumers. While increases in drug overdose deaths have largely been driven by illicitly manufactured fentanyls and their variable concentrations within illicit drug supplies [1, 2], there has been a recent rise in xylazine-positive overdose deaths [18]. Despite these concerning trends, active surveillance of chemical contaminants in illicit controlled substances has historically been limited to medical examiner reports and law enforcement seizures, which investigate chemical compositions of substances only after adverse events have occurred. A growing number of drug-checking programs are emerging; however, these generally only receive batches of illicit drugs after community members witness unusual reactions, and they do not include bacteriologic assessments. This status quo may miss critical surveillance of harmful adulterants and pathogens until after adverse outcomes are widely recognized within the local community. Syringe residue analysis offers an antidote to many of these concerns by offering a wider view of compounds and pathogens circulating within an injection drug use community [19].

To evaluate the potential bacterial and chemical contaminants PWID are exposed to in the local illicit drug supply, we used mass spectrometry, along with microbiologic cultures of syringe residue from discarded drug use equipment collected by harm reduction organizations in St Louis, Missouri, over a 12-month time period from 2022 to 2023.

## METHODS

### Sample Collection

Discarded injection drug use needles and syringe equipment was collected from sharps containers provided by street medicine outreach and harm reduction teams from Street Med STL, a volunteer street medicine and harm reduction organization that provides care to unhoused persons who use drugs. Outreach occurred weekly over a 12-month time period at 5 different sites in St Louis, Missouri. Sharps were only collected if properly disposed of in a sharps container (no loose refuse was obtained). Once collected, sharps containers were transferred to research laboratories at Washington University in St Louis. Forceps were used to safely remove only capped syringes, in order to reduce the chance that any pathogens cultured in this analysis could stem from the external environment within the sharps container. All other injection drug use equipment was discarded and not included in the analysis. Time from collection by Street Med STL to processing by laboratory workers for bacterial culture analysis was within 1 week.

### Bacterial Culturing and Analysis

The capped needle-syringe combinations were cultured by removing the plunger on the back of the syringe and inserting a sterile culture swab (BD ESwab), which was plated on a variety of culture media and culture conditions including blood agar, MacConkey agar, *Escherichia coli* Coliform Selective Agar (Millipore Sigma), and mannitol salt phenol red agar (Millipore Sigma), and incubated under both routine and anaerobic conditions at 37°C. Unique colony morphotypes from individual plates were identified and broth enrichment was performed in Luria-Bertani broth, THY medium (Todd-Hewitt broth with 2% yeast extract), or tryptic soy broth. Genomic DNA was extracted from pure cultures using the Puregene Yeast/Bacteria Genome Purification Kit (Qiagen, Hilden, Germany). As *S aureus* colonies could be immediately identified due to their coloration on mannitol salt phenol red agar, relative abundance of these was also recorded.

### Whole Genome Sequencing

Whole genomic DNA was submitted for shotgun sequencing to an average depth of 1.8 million reads (100–200× genome coverage) on the NovaSeq 6000. Raw sequencing reads were trimmed of adaptors using trimmomatic, human DNA contamination was removed with Deconseq, files were repaired with bbmap, and genomes were assembled with Unicycler. CheckM and Quast were used to check genome statistics. Assemblies were retained if they were >90% complete with <5% contamination. Mash was used to assign taxonomy.

### Liquid Chromatography–Mass Spectrometry

Mass spectrometry analysis was performed for a subset of 100 syringes. Swabs were inserted into 1 mL of mass spectrometry–grade water for 5 minutes and then removed, and the solution was filtered through a 40 µm mesh followed by 2000*g* centrifugation for 12 minutes. Supernatant was immediately aliquoted into cryovials and stored at −20°C until ready for experimental analysis. Mass spectrometry–grade methanol (mass spectrometry grade, Sigma-Aldrich) was used as a control.

Untargeted mass spectrometry was performed using an Orbitrap ID-X high-resolution accurate mass spectrometer coupled to a Vanquish ultra high performance liquid chromatography mass spectrometer (Thermo Scientific), as described in the Supplementary Materials (Mass Spectrometry Methods). In brief, separation was performed with a reverse phase Waters Acquity HSS T3 column (100 Å, 1.8 µm, 2.1 mm × 150 mm) maintained at 30°C with a constant flow rate of 0.4 mL/minute. Proportions of mobile phase buffers A (0.1% formic acid in H_2_O) and B (0.1% formic acid and 90% acetonitrile in H_2_O) were varied according to the following gradient: constant 2% B from 0 to 2 minutes, linear gradient to 27% B at 9 minutes, linear gradient to 98% B at 16 minutes, hold until 18 minutes, back to 2% B at 19 minutes, and equilibration at 2% B until 22 minutes. MS1 scans were collected between 100 and 1000 m/z using the Orbitrap detector with quadrupole isolation and resolution set to 60 000. Further detailed mass spectrometry parameters can be found in the Supplementary Materials (Mass Spectrometry Methods). Identification of features was performed using the automated AcquireX protocol (Thermo Scientific) with comparison to the mzCloud database (Thermo Scientific), as further described in the Supplementary Materials (Mass Spectrometry Methods).

Residue from all 100 syringes was analyzed using multiple reaction monitoring (MRM) targeted mass spectrometry to determine the presence or absence of common illicit substances and adulterants listed in the Supplementary Materials (Mass Spectrometry Methods) and Supplementary Table 1.

MRM was performed using an AB Sciex 4000 QTrap triple-quadrupole mass spectrometer coupled to a Shimadzu UFLC. In brief, separation was performed with a reverse phase Supelco Ascentis Express Phenyl-Hexyl (90 Å, 2.7 µm, 10 cm × 2.1 mm) at room temperature. Total flow rate was set to 0.35 mL/minute. Proportions of mobile phase buffers A (0.1% formic acid in H_2_O) and B (0.1% formic acid and 90% acetonitrile in H_2_O) were varied according to the following gradient: constant 2% B from 0 to 1 minutes, linear gradient to 35% B at 23 minutes, linear gradient to 98% B at 24 minutes, hold until 27 minutes, back to 2% B at 28 minutes, and equilibration at 2% B until 31 minutes. Samples were analyzed in positive ion mode, with detailed parameters described in the Supplementary Materials (Mass Spectrometry Methods). US Drug Enforcement Administration–exempt standards were purchased when possible for comparison. Peak calls for presence/absence were manually verified by 3 authors.

### Patient Consent Statement

The Washington University School of Medicine Human Research Protection Office designated this activity as non–human subjects research as no identifiable information was collected, and analyses were limited to discarded injection equipment.

## RESULTS

### Microbiologic Survey of Syringe Residue

A total of 160 syringe residue samples from injection drug use equipment were analyzed. Bacteria were cultured from 94 of the 160 syringes analyzed (58.75%). Polymicrobial growth was common and was observed in 38 of the 160 samples (23.75%) analyzed. The list of all bacterial species isolated is shown in Table 1. Medically important pathogens cultured from syringe residue included *Acinetobacter* spp, *Bacillus cereus*, *Enterobacter* spp, *Klebsiella* spp, *Pseudomonas* spp, *Serratia marcescens*, *S aureus*, and coagulase-negative staphylococci. *Bacillus cereus* was the most common pathogen present, observed in 20.6% of syringe residues, followed closely by *S aureus* at 18.8%.

**Table 1.** Bacterial Pathogens Cultured From Syringe Residue of Discarded Injection Drug Use Paraphernalia, Listed in Order of Prevalence

Pathogen	Prevalence
Bacillus cereus	33 (20.6%)
Staphylococcus aureus	30 (18.8%)
Bacillus altitudinis	14 (8.8%)
Bacillus velezensis	11 (6.9%)
Priestia megaterium	10 (6.3%)
Bacillus safensis	9 (5.6%)
Pseudomonas aeruginosa	5 (3.1%)
Bacillus flexus	4 (2.5%)
Bacillus licheniformis	4 (2.5%)
Exiguobacterium acetylicum	4 (2.5%)
Planococcus citreus	4 (2.5%)
Bacillus pumilus	2 (1.3%)
Brucella intermedia	2 (1.3%)
Acinetobacter variabilis	1 (0.6%)
Aerococcus urinaeequi	1 (0.6%)
Microbacterium paraoxydans	2 (1.3%)
Microbacterium sp	2 (1.3%)
Staphylococcus capitis	2 (1.3%)
Bacillus aryabhattai	1 (0.6%)
Bacillus nealsonii	1 (0.6%)
Brachybacterium paraconglomeratum	1 (0.6%)
Brevibacillus centrosporus	1 (0.6%)
Brevibacillus parabrevis	1 (0.6%)
Cellulosimicrobium cellulans	1 (0.6%)
Cytobacillus sp	1 (0.6%)
Enterobacter hormaechei	1 (0.6%)
Enterobacter sp	1 (0.6%)
Klebsiella michiganensis	1 (0.6%)
Kocuria carniphila	1 (0.6%)
Kocuria polaris	1 (0.6%)
Leclercia adecarboxylata	1 (0.6%)
Lysinibacillus boronitolerans	1 (0.6%)
Paenibacillus polymyxa	1 (0.6%)
Paenibacillus sp	1 (0.6%)
Pseudomonas oryzihabitans	1 (0.6%)
Pseudomonas otitidis	1 (0.6%)
Serratia marcescens	1 (0.6%)
Sphingomonas dokdonensis	1 (0.6%)
Staphylococcus epidermidis	1 (0.6%)
Staphylococcus hominis	1 (0.6%)
Staphylococcus xylosus	1 (0.6%)

As *S aureus* is among the pathogens causing the highest burden of disease in PWID, and colonies could be immediately identified due to their coloration on mannitol salt phenol red agar, the relative abundance of *S aureus* colonies was also recorded. Among the 30 syringe residues where *S aureus* was cultured, 7 samples had rare *S aureus* with between 1 and 10 colony-forming units (CFU) per plate noted when swabs were directly plated from syringe residue onto mannitol salt phenol red agar, 10 samples showed moderate growth with between 10 and 100 CFU per plate, and 13 samples grew abundant *S aureus* showing a confluent lawn of *S aureus* too numerous to individually enumerate colonies.

### Mass Spectrometry Analysis of Syringe Residue

One hundred syringes from sharps containers collected by Street Med STL during harm reduction outreach were analyzed by liquid chromatography–MRM over the 12-month timespan. To detect potential illicit substances and chemical adulterants in the drug supply, untargeted mass spectrometry was performed on syringes collected during or before January 2023. Untargeted mass spectrometry revealed a wide range of illicit substances and their associated metabolites. These included fentanyl; fentanyl metabolites, analogues, and precursors; methamphetamines; cocaine; benzoylecgonine; heroin; 4-ANPP; tramadol; methcathinone; and ephedrine. Methcathinone, tramadol, and ephedrine were only detected in trace amounts in a small number of syringes and so were not included in targeted scans. Targeted LC-MRMs were developed for the most common illicit substances identified by untargeted mass spectrometry. Of the 100 syringes analyzed by targeted LC-MRMs, 98 of 100 (98%) contained residue of either methamphetamines, fentanyl, or both, confirming that syringes collected for this project were previously used for injection of illicit drugs. Of the 98 syringes that contained drug residue, targeted LC-MRMs detected methamphetamine in 97 (99%) syringes, 1 or more opioids were detected in 83 (85%) syringes, and cocaine was detected in 56 (57%) syringes (Table 2). Among the 83 opioid-positive samples, 83 of 83 (100%) contained fentanyl, 13 of 83 (16%) contained acetyl-fentanyl, and 23 of 82 (28%) contained heroin or the heroin metabolite 6-acetylmorphine.

**Table 2.** Illicit Drugs and Medically Important Adulterants Detected in Syringe Residue

Drug Group	Substance	Prevalence (N = 100 Samples)
Stimulants	Benzoylecgonine	39 (39%)
	Cocaine	56 (56%)
	Methamphetamine	97 (97%)
Opioids	Fentanyl (including metabolites, analogues, and precursors)	83 (83%)
	4-ANPP	76 (76%)
	Heroin	13 (13%)
	6-Acetylmorphine	22 (22%)
	Acetylfentanyl	13 (13%)
Adulterants	Diphenhydramine	83 (83%)
	Caffeine	59 (59%)
	Lidocaine	66 (66%)
	Levamisole	1 (1%)
	Quinine	52 (52%)
	Xylazine	58 (58%)

Initial untargeted mass spectrometry had also revealed a range of medically significant adulterants including caffeine, diphenhydramine, lidocaine, quinine, and xylazine. Standards for medically significant adulterants were obtained for caffeine, diphenhydramine, lidocaine, quinine, and xylazine in order to verify their identity and prevalence within the samples. In addition, a standard for levamisole was purchased as it is a published chemical adulterant. Targeted liquid chromatography–mass spectrometry (LC-MS) runs confirmed that the presence of these chemical adulterants was common in the 98 syringes containing drug residue, including caffeine (59/98 [60%]), diphenhydramine (83/98 [85%]), lidocaine (66/98 [67%]), quinine (52/98 [53%]), and xylazine (58/98 [59%]). Levamisole was uncommon and was only detected in 1 of 98 (1%) syringes containing drug residue where it was found in combination with cocaine. The prevalence of xylazine and quinine among fentanyl-containing syringe residues remained relatively stable over the project period (Supplementary Figure 1).

## DISCUSSION

The data presented in this analysis demonstrates that the illicit drug supply in Missouri is contaminated with numerous chemical adulterants, and that a variety of different bacterial pathogens are present in syringe residue. There are several potential points at which bacteria may have been introduced into syringe residue: (1) during the manufacturing process; (2) during the distribution and repackaging within the supply chain; (3) from solvents used in the preparation of illicit drugs immediately prior to injection; (4) during drug mixing and handling; (5) from skin contamination during the injection process; or (6) resulting from draw-back into the syringe if PWID are bacteremic.

Contaminated illicit drugs and unsafe drug preparation practices are a well-described cause of infections in PWID. Prior work conducted in the wake of local outbreaks has cultured *B cereus* and *Clostridium* spp from confiscated heroin supplies, offering confirmation of the role of microbial contamination of heroin in causing injection drug use–associated infections [15, 20]. More recently, Kasper et al evaluated previously used cookers for the presence of *S aureus* and found that 14% of cookers/filters used for injection of opioids were contaminated, raising the possibility that both illicit drugs and drug preparation equipment could serve as sources of infection [21].

Outbreaks of infections linked to contaminated medications can cause severe disease and even deaths. In the US, these have been largely associated with pharmacy-compounded sterile preparations with the most common practices associated with contamination, including compounding originating from nonsterile ingredients and repackaging of already sterile products [22–24]. Breaches in aseptic processing and deficiencies in sterilization procedures or in sterility/endotoxin testing are frequently associated with these infectious outbreaks [23]. The unregulated nature of illicitly manufactured fentanyls and stimulants allows for many of these nonsterile practices, increasing the risk of medication-associated outbreaks among PWID.

Perhaps the most concerning finding in this study is the overall stability and relative abundance of pathogenic bacterial species in syringe residue, highlighting the potential for transmission of bacterial pathogens through sharing of drug use equipment. While many of the bacterial species identified here are environmental organisms not known to cause infections in immunocompetent hosts, a number of important human pathogens that cause both serious skin and soft tissue infections and infective endocarditis in PWID were identified. These included *Acinetobacter* [25], *B cereus* [26, 27], *Pseudomonas aeruginosa* [28], *S marcescens* [29], *S aureus* [11], and coagulase-negative staphylococci [7, 30].

Despite a shift in the type of illicit drugs used by PWID from heroin to illicitly manufactured fentanyls and methamphetamines, pathogens isolated from syringe residue in this analysis mirror those previously identified in microbiologic studies of heroin performed in the 1970s, 1980s, and more recently in 2002 [15]. Microflora were composed primarily of endospore-forming bacteria (*Bacillus* and *Paenibacillus* spp) as well as staphylococci. Endospore-forming bacteria are frequently present in soil in the environment; and researchers had previously hypothesized that these could be the result of the initial heroin manufacturing process as it was derived from poppies [15]. However, given their presence in the syringe residue of illicitly manufactured fentanyls and stimulants, the source seems more likely to be post-processing contact with the environment or injectors. While staphylococci do not produce endospores, they are known to persist on fomites [31]. All *Staphylococcus* spp isolated in this analysis are primary colonizers of human skin and thus 1 potential source could be direct contact with the skin flora of the drug producers, processors, distributors, or PWID. Their presence and relative stability in syringe residue along with needle and syringe sharing practices may explain their transmission within the injection drug use network [9, 10]. By focusing on syringe residue from used, capped, needle–syringe combinations, this analysis also captures any adulterants or pathogens introduced by solvents or drug preparation. This may explain the presence of pathogens such as *Pseudomonas* and *Serratia*, which are more typically associated with contaminated water and could reflect a lack of access to sterile water for drug preparation.

Several species that commonly cause bloodstream infections in PWID, including streptococci and oral anaerobes, were notably absent from this analysis. Interestingly, they have not been isolated in any investigations of heroin microflora in the past [15]. The ongoing absence of these pathogens may be due to their limited stability on fomites and in syringe residue, or may reflect that these pathogens are introduced into patients only after illicit drugs are prepared, such as through licking needles. If introduced through licking needles, oral streptococci and other oral anaerobes would not be expected to be present in syringe residue and would be only present on the outer surface of the needle itself. Needles were not cultured in this analysis, both to reduce the risk to laboratory workers and to limit the potential that any positive cultures could be the result of handling after injection drug use, or due to environmental pathogens within the sharps container.

Untargeted mass spectrometry analysis of syringe residue revealed the presence of a number of medically important chemical adulterants, which was confirmed with the use of chemical standards by targeted mass spectrometry. More than half of syringes analyzed by LC-MS contained the adulterant xylazine, which has been increasing in prevalence in illicit drug supplies across the US [18, 32] and is associated with severe ulcers and nonhealing wounds [33]. Our analysis also revealed a significant number of other adulterants in syringe residue including caffeine, diphenhydramine, lidocaine, and quinine. The ongoing presence of quinine is important for clinicians to be aware of as overdoses of quinine can result in both cardiovascular toxicity and platelet disorders, including arrhythmias, chest pain, and thrombocytopenia [34]. Although levamisole has been historically documented as a common chemical adulterant of cocaine, of medical importance due to its ability to cause severe vasculitis [35], levamisole was present in only 1 sample. Most syringes demonstrated multiple illicit drugs, suggesting either unintentional poly-drug use or that syringes may have been used on multiple occasions for injecting multiple different drugs. Compared to another recent study analyzing syringe residue from needle-exchange syringes in Washington, District of Columbia (DC) [19], our study found a much higher proportion of syringes positive for methamphetamine, cocaine, and poly-drug contamination. We speculate that may be due to the direct collection of syringes from street encampments, which may represent a population engaging in higher-risk injection behaviors or using multiple substances.

Syringe access programs are evidence-based harm reduction interventions that can provide PWID with safe disposal and exchange of used syringes for new syringes and other supplies [36, 37]. Currently, syringe access programs operate in 44 states as well as Washington, DC and Puerto Rico; however, only 38 states have officially legalized syringe access program operation and Missouri is not among these [38]. The presence of multiple chemical adulterants and bacterial pathogens within syringe residue from St Louis, Missouri, suggests an urgent need for broader and more timely drug-checking services, as well as wider access to sterile harm reduction supplies including those that might be provided by syringe access programs.

This report has several important limitations. As injection drug use equipment was collected through outreach, individual PWIDs may have contributed multiple syringes at any given time. To reduce the likelihood of any individual sample or PWID predominating our results, we collected samples during routine outreach efforts at 5 different sites over a 12-month timespan. Furthermore, by collecting discarded injection drug use equipment over multiple sites and dates during routine harm reduction activities, these data present a more agnostic picture of the bacterial and chemical contaminants in the local drug supply compared to drug use samples submitted to drug-checking programs, which may represent only those samples that individual participants feel are causing unusual harms. Another important limitation is that syringe sharing and reuse is common, particularly among unhoused PWID. This is likely the reason for the high preponderance of syringe residue samples that contained multiple illicit drugs. This analysis cannot distinguish between poly-substance use, serial use of individual substances by an individual person, or syringe sharing.

## CONCLUSIONS

Analysis of syringe residue from discarded drug use equipment used by PWID demonstrates both chemical and biological contaminants including medically important pathogens. These data illustrate the strength of syringe residue surveillance to monitor drug trends and how this method can be used to detect emerging chemical contaminants and pathogens in the future.

## Supplementary Data

Supplementary materials are available at *Open Forum Infectious Diseases* online. Consisting of data provided by the authors to benefit the reader, the posted materials are not copyedited and are the sole responsibility of the authors, so questions or comments should be addressed to the corresponding author.

## Supplementary Material

ofad628_Supplementary_Data

## Notes

***Author contributions*.** L. R. M., J. A. W., and J. P. H. conceptualized and designed the study. D. J. S. and L. Z. performed the genomic analysis and sequencing. L. R. M., J. A. W., and E. J. conducted all microbiologic studies. J. A. W. and J. L. R. performed all mass spectrometry. N. S. N. and R. J. coordinated harm outreach and interpretation of local drug use trends. All authors had full access to all data in the study and take responsibility for the integrity of the data and the accuracy of the data analysis. All authors contributed to the writing and critical revision of the report. All authors contributed to the data acquisition, data analysis, or data interpretation and reviewed and approved the final version.

***Patient consent*.** This study was designated non–human subjects research by the Washington University Institutional Review Board before any research activities were performed.

***Financial support*.** This work was supported in part by funds from the Institute for Public Health at Washington University in St. Louis (to N. S. N.) and the Longer Life Foundation (to L. R. M.). D. J. S. is supported by the Doris Duke Charitable Foundation (Physician Scientist Fellowship grant number 2021081) and the National Institute of Allergy and Infectious Diseases (award number K08 KAI159384). R. J. is supported by the National Institute on Drug abuse (grant number NIDAK12DA050607) and J. P. H. is supported by the National Institute of Diabetes and Digestive and Kidney Diseases (grant number R01DK125860).
