# Supplementary material for: Everything but the Kitchen Sink: An Analysis of Bacterial and Chemical Contaminants Found in Syringe Residue From People Who Inject Drugs
Source: Open Forum Infect Dis. 2023 Dec 11;11(1):ofad628. doi: 10.1093/ofid/ofad628 (PMC10766411; doi:10.1093/ofid/ofad628)
Supplement: ofad628_Supplementary_Data [file ofad628_supplementary_data.zip › Syringe Residue paper Supplemental methods.docx]

**Supplemental Mass Spectrometry methods**

**Untargeted Mass Spectrometry**

Untargeted mass spectrometry was performed using an Orbitrap ID-X high-resolution accurate mass spectrometer coupled to a Vanquish UHPLC (Thermo Scientific). Sample vials were placed in an autosampler and held at 14 °C. Injection volume for all samples was 5 µL. Needle residues were separated using a Waters Acquity HSS T3 column (100 Å, 1.8 µm, 2.1 mm x 150 mm) maintained at 30 °C with a constant flow rate of 0.4 mL/min. Proportions of mobile phase buffers A (0.1% formic acid in H_2_O) and B (0.1% formic acid and 90% acetonitrile in H_2_O) were varied according to the following gradient: constant 2% B from 0 to 2 minutes, linear gradient to 27% B at 9 minutes, linear gradient to 98% B at 16 minutes, hold until 18 minutes, back to 2% B at 19 minutes, and equilibration at 2% B until 22 minutes. Samples were ionized by an H-ESI ion source with spray voltage alternating between +3400 V and -2400 V for positive- and negative-ion scans, respectively. Sheath gas was set to 50, auxiliary gas to 10, and sweep gas to 1. The ion transfer tube was held at 325 °C and the vaporizer at 350 °C. MS1 scans were collected between 100-1000 m/z using the Orbitrap detector with quadrupole isolation and resolution set to 60,000. Maximum injection time was adjusted dynamically with a normalized automatic gain control (AGC) target of 100%. RF lens was set to 45%. Cycle time was set to 1 second.

To assist identification of needle residue components, we used the automated AcquireX protocol (Thermo Scientific) to collect MS2 and MS3 spectra of compounds detected in the pooled quality control specimen. This specimen was injected and separated four times each for positive and negative ion modes as described above. MS1 scans were collected as described above except that the RF lens was set to 35%. MS2 spectra were collected at most 3 times every 3 seconds for MS1 ions exceeding an intensity of 20,000. MS2 scans were collected in quadrupole isolation mode with a 1.5 Da isolation window and automatic scan range. The MS1 ion was fragmented in assisted HCD mode using collision energies of 20, 35, 50, 75, and 100%. Fragments were detected in the Orbitrap at 15,000 resolution with a maximum injection time of 22 ms and a normalized ACG target of 100%. MS3 scans were collected on the 3 most intense MS2 ions with intensities exceeding 5,000. MS2 ions between 0.5 Da below and 3 Da above the precursor ion were not considered for MS3 scans. MS3 scans were collected in quadrupole isolation mode with 1.5 Da MS1 and 2 Da MS2 isolation windows and automatic scan range. MS2 ions were fragmented in fixed energy CID mode using a collision energy of 30%, 10 ms activation time, and activation Q of 0.25. Fragments were detected in the ion trap using the rapid scan rate, maximum injection time of 50 ms, and normalized AGC target set to 200%.

Data was collected using Xcalibur software and both MS1 and MS2/3 data were aligned using Compound Discoverer 3.3 (Thermo Scientific). Detailed interrogation of spectral data and prediction of molecular formulas was performed using FreeStyle and MassFrontier software (Thermo Scientific), and comparison to spectral databases using mzCloud (Thermo Scientific).

**Targeted MRM scans**

Targeted mass spectrometry was performed using an AB Sciex 4000 QTrap triple-quadupole mass spectrometer coupled to a Shimadzu UFLC. Separation was performed with a Supelco Ascentis Express Phenyl-Hexyl (90 Å, 2.7 µm, 10 cm x 2.1 mm, Cat# 53336-U) at room temperature. Total flow rate was set to 0.35 mL/min. Proportions of mobile phase buffers A (0.1% formic acid in H_2_O) and B (0.1% formic acid and 90% acetonitrile in H_2_O) were varied according to the following gradient: constant 2% B from 0 to 1 minutes, linear gradient to 35% B at 23 minutes, linear gradient to 98% B at 24 minutes, hold until 27 minutes, back to 2% B at 28 minutes, and equilibration at 2% B until 31 minutes. Samples were analyzed in positive ion modes using a Turbo V electrospray ionization source with ion spray voltage set to 4500 V and heater temperature at 600 °C. Ion source gas 1 and 2 were set to 45 and curtain gas set to 35. Collision gas flow was set to “low”. Molecular ions were monitored in Multiple Reaction Monitoring (MRM) mode with declustering potential (DP) and collision energy (CE) as shown below. For all metabolites, EP and CXP were set to 10 and 12 V, respectively.

| **Compound** | **Q1** | **Q3** | **DP (V)** | **CE (V)** | **Source(s)** | **Notes** |
| --- | --- | --- | --- | --- | --- | --- |
| levamisole | 205 | 178 | 55 | 33 | Gao et al 2021 | DP/CE manually determined for our instrumentation |
| cocaine | 304.1 | 182.1 | 36 | 27 | Herrin, McCurdy, Wall 2005 | |
| methamphetamine | 150.1 | 91 | 31 | 23 | Herrin, McCurdy, Wall 2005 | |
| fentanyl | 337.2 | 105 | 60 | 60 | Herrin, McCurdy, Wall 2005; Strayer et al 2018 | A Q3 ion of 105 by Strayer et al 2018 performed better on our instrumentation than 188 (used by Herrin, McCurdy, and Wall 2005) |
| xylazine | 221.1 | 164.1 | 68 | 35 | Li et al 2021 | |
| caffeine | 195.1 | 138 | 51 | 27 | Herrin, McCurdy, Wall 2005 | |
| quinine | 325.2 | 172 | 51 | 47 | Herrin, McCurdy, Wall 2005 | |
| lidocaine | 235.2 | 86 | 31 | 25 | Herrin, McCurdy, Wall 2005 | |
| diphenhydramine | 256.2 | 167 | 16 | 21 | Herrin, McCurdy, Wall 2005 | |
| heroin | 370.2 | 165 | 90 | 60 | Agilent 2017 | DP/CE manually determined for our instrumentation; Q3 165 ion outperformed 268.1 on our instrumentation |
| 6-acetylmorphine | 328.1 | 165 | 61 | 55 | Herrin, McCurdy, Wall 2005 | |
| acetylfentanyl | 323 | 105 | 30 | 20 | Strayer et al 2018 | DP determined manually |
| 4-ANPP | 281.4 | 105.1 | 30 | 36 | Strayer et al 2018 | DP determined manually |
| benzoylecgonine | 290.1 | 168 | 36 | 27 | Herrin, McCurdy, Wall 2005 | |

Sources for MRM parameters:

Gao P, Zhang P, Guo Y, He Z, Dong Y, Tang Y, Guan F, Zhang T, Xie K. Determination of Levamisole and Mebendazole and Its Two Metabolite Residues in Three Poultry Species by HPLC-MS/MS. Foods. 2021 Nov 17;10(11):2841. doi: 10.3390/foods10112841. PMID: 34829122; PMCID: PMC8624778.

Herrin GL, McCurdy HH, Wall WH. Investigation of an LC-MS-MS (QTrap) method for the rapid screening and identification of drugs in postmortem toxicology whole blood samples. J Anal Toxicol. 2005 Oct;29(7):599-606. doi: 10.1093/jat/29.7.599. PMID: 16419388.

Strayer KE, Antonides HM, Juhascik MP, Daniulaityte R, Sizemore IE. LC-MS/MS-Based Method for the Multiplex Detection of 24 Fentanyl Analogues and Metabolites in Whole Blood at Sub ng mL-1 Concentrations. ACS Omega. 2018 Jan 31;3(1):514-523. doi: 10.1021/acsomega.7b01536. Epub 2018 Jan 17. PMID: 29399650; PMCID: PMC5793031.

Xiaoqin Li, Zhijuan Fang, Xinye He, Shuqin Zhang, Hongliu Ding & Hu Ye (2021) Optimization of 204 veterinary drug residues method and establishing their mass spectrum library, International Journal of Food Properties, 24:1, 1658-1680, DOI: 10.1080/10942912.2021.1986524

Agilent Technologies. Comprehensive LC/MS Analysis of Opiates, Opioids, Benzodiazepines, Amphetamines, Illicits, and Metabolites in Urine. https://www.agilent.com/cs/library/applications/5991-1667EN.pdf. E-published January 25, 2017.
