# Supplementary figures and images for: Everything but the Kitchen Sink: An Analysis of Bacterial and Chemical Contaminants Found in Syringe Residue From People Who Inject Drugs
Source: Open Forum Infect Dis. 2023 Dec 11;11(1):ofad628. doi: 10.1093/ofid/ofad628 (PMC10766411; doi:10.1093/ofid/ofad628)

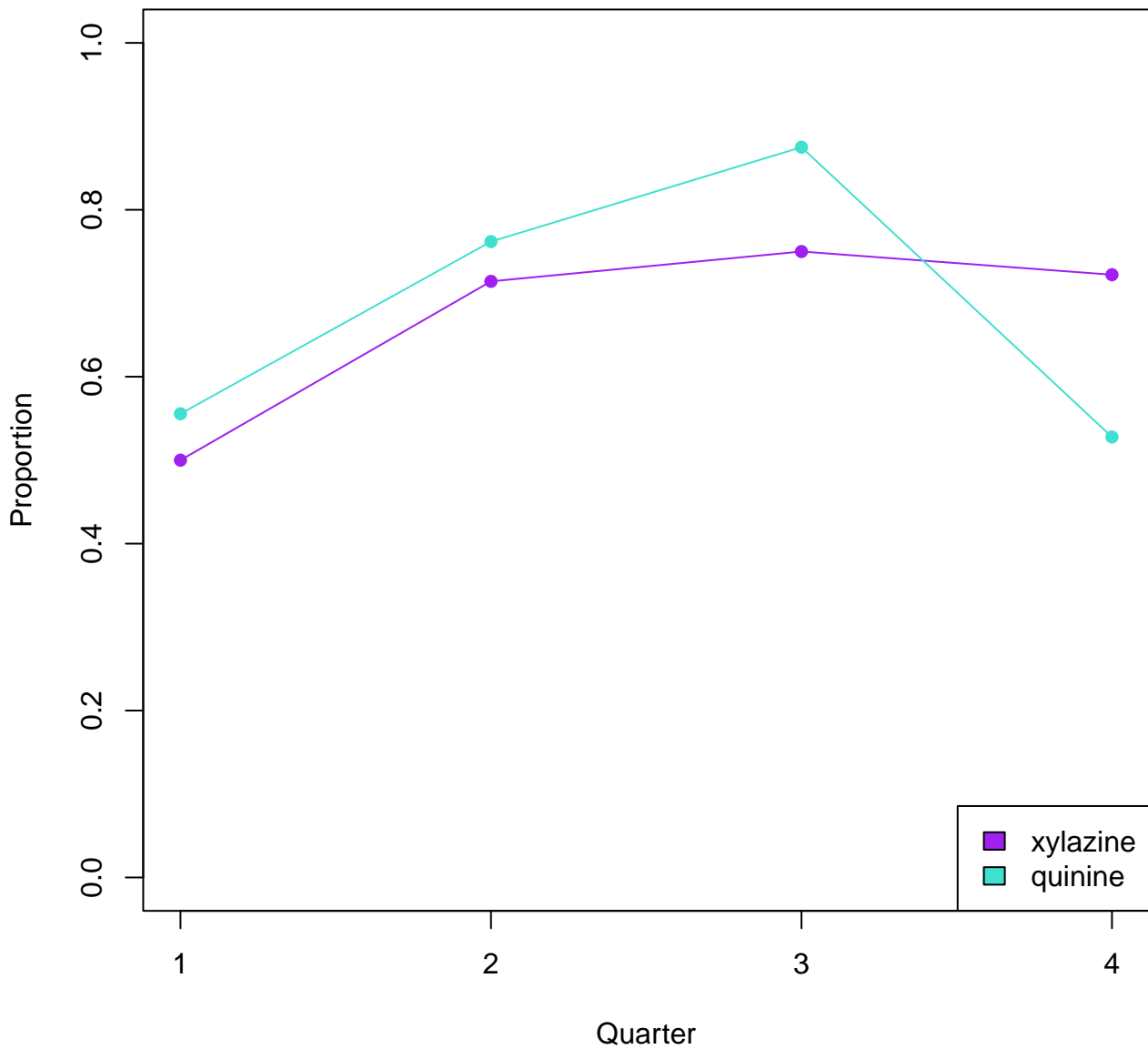

Supplement: ofad628_Supplementary_Data [file ofad628_supplementary_data.zip › Supplemental Figure 1.pdf]
